# Secular trends in grip strength among Korean adults according to socioeconomic factors: the 2014-2022 Korea National Health and Nutrition Examination Survey

**DOI:** 10.4178/epih.e2025074

**Published:** 2025-12-16

**Authors:** Harim Choe, Hoyong Sung, Yeon Soo Kim

**Affiliations:** 1 Department of Physical Education, Korea Military Academy, Seoul, Korea; 2 Department of Physical Education, Seoul National University College of Education, Seoul, Korea; 3 Institute of Sports Science, Seoul National University, Seoul, Korea

**Keywords:** Trend analysis, Hand strength, Social factors, Economic factors

## Abstract

**OBJECTIVES:**

Muscle strength is a key indicator of overall health, and its decline has been linked to increased morbidity and mortality. Socioeconomic factors may contribute to disparities in this decline. Therefore, this study aimed to examine trends in muscle strength and to identify groups with lower muscle strength according to socioeconomic variables.

**METHODS:**

We analyzed data from the cross-sectional Korea National Health and Nutrition Examination Survey (KNHANES), including 34,080 adults. Multivariable linear regression analyses were conducted, adjusting for socioeconomic and health-related covariates, and KNHANES survey weights were applied to obtain nationally representative estimates that accounted for the complex sampling design.

**RESULTS:**

Overall, mean grip strength significantly decreased from 2014 to 2022 in both males (from 43.45±0.24 to 41.59± 0.29 kg) and females (from 26.48±0.15 to 24.94±0.13 kg). This trend was consistently observed across all covariate strata, except for individuals aged over 70 years and those in the green-collar occupational group. In 2022, grip strength was lower among individuals with the lowest education level (26.45±0.34 vs. 34.75±0.32 kg in the undergraduate group) and the lowest household income level (29.59±0.45 vs. 34.53±0.35 kg in the highest income group), as well as among unemployed individuals (29.36± 0.30 vs. 37.00±0.47 kg in the blue-collar group), compared with their higher socioeconomic counterparts.

**CONCLUSIONS:**

These findings provide descriptive evidence of grip strength trends and socioeconomic disparities in Korea. They may serve as baseline information to guide future longitudinal studies and inform public health strategies.

## GRAPHICAL ABSTRACT

[Fig f3-epih-47-e2025074]

## Key Message

This study demonstrates secular changes in grip strength among Korean adults and reveals persistent socioeconomic disparities by education, income, and occupation. These findings provide descriptive evidence to help identify population groups with lower grip strength.

## INTRODUCTION

As a society ages, the ability to live independently in later life becomes increasingly important. Independence in daily life can be assessed using activities of daily living (ADL) and instrumental activities of daily living (IADL). ADL evaluates fundamental tasks such as transferring, feeding, and dressing, whereas IADL encompasses more complex, community-based activities compared with those assessed by ADL [1]. Higher ADL or IADL scores indicate fewer limitations in performing daily activities. Among the components of physical fitness required for daily functioning, muscle strength is particularly important, and a decrease in muscle strength has been associated with lower ADL and IADL scores [2]. Furthermore, several studies have reported that declining muscle strength is associated with reduced quality of life, deteriorating mental health, and an increased risk of falls [3-5].

Muscle strength can be measured using various methods, including isokinetic tests that require expensive equipment, 1-repetition maximum tests, and measurements of grip strength and leg extension. Among these, grip strength testing assesses the ability to grasp objects by hand through coordinated finger function. Grip strength is known to reflect overall muscle strength and is considered a more efficient measure than other strength tests because of its low cost and simplicity [6,7]. Given these advantages, grip strength is commonly used as a key variable in epidemiological studies. Although the importance of preventing declines in muscle strength and the utility of grip strength testing are well recognized, few studies have evaluated long-term trends in grip strength [8,9]. These studies consistently reported an overall decrease in grip strength during their respective study periods. For example, Kang et al. [10] examined grip strength trends in Korean adolescents aged 10-18 years from 2014 to 2017 and observed a slight downward trend in both boys and girls (boys, 33.32±0.81→31.60±0.61 kg; girls, 23.38±0.48→21.92±0.31 kg). However, that study focused solely on Korean adolescents, and to date, no analysis has evaluated trends in grip strength among Korean adults. Muscle strength typically peaks in early adulthood and declines with age, and low grip strength in adulthood is a well-established predictor of morbidity, disability, and mortality. Understanding trends in adult grip strength is therefore critical for public health monitoring.

Muscle strength can be improved through regular participation in muscle-strengthening exercise (MSE). Nevertheless, in epidemiological research, grip strength reflects not only individual behaviors but also broader social determinants of health. Socioeconomic conditions—such as education, household income, and occupation—can influence health behaviors and access to resources, thereby contributing to disparities in muscle strength across populations. Several studies have reported that individuals with lower socioeconomic status exhibit weaker grip strength [11-14]. Based on this rationale, the present study aimed to examine temporal trends in grip strength among Korean adults and to identify socioeconomic groups at higher risk of low muscle strength using nationally representative data from the Korea National Health and Nutrition Examination Survey (KNHANES).

## MATERIALS AND METHODS

### Study design

**Figure 1.**
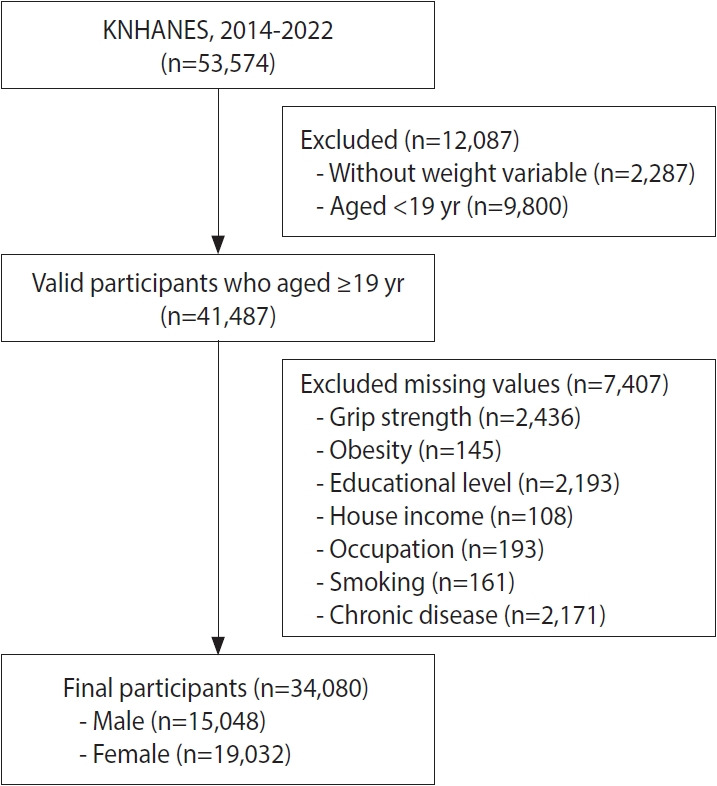
Flow chart of participant selection. KNHANES, Korea National Health and Nutrition Examination Survey.

The KNHANES, conducted by the Korea Disease Control and Prevention Agency (KDCA), assesses health status, chronic diseases, and nutritional intake in the Korean population. The KNHANES uses the most recent population and housing census as the basic sampling frame, thereby ensuring national representativeness. The survey was conducted every 3 years from 1998 and has been conducted annually since 2007. Grip strength assessment was introduced in 2014; however, there were insufficient grip strength data for 2020-2021 because of the coronavirus disease 2019 (COVID-19) pandemic. Therefore, the present study excluded data from this period, and a total of 53,574 participants were initially included. To ensure data representativeness, individuals without survey weights (n=2,287) were excluded because they could not contribute to nationally representative estimates. In addition, children and adolescents (n=9,800) were excluded to focus on adults, and participants with missing data on grip strength or covariates were excluded by listwise deletion (n=7,407). The final analytic sample included 34,080 adults (Figure 1).

### Measurement of grip strength

The KNHANES has measured grip strength using a digital grip strength dynamometer (TKK 5401; Takei, Tokyo, Japan) to assess muscle strength among participants aged 10 years and older. Prior to the assessment, instructors conducted a screening survey to identify individuals who might have limitations affecting grip strength measurement. Participants without such limitations were instructed to adjust the handle so that it formed a 90-degree angle with the second finger joint. While standing, they were instructed to keep their elbows and wrists unbent and to maintain a slight distance between their arms and torso to avoid contact. In accordance with these instructions, participants were asked to grasp the dynamometer to the best of their ability for 3 seconds. After assessing both hands, participants were given a 60-second rest before each subsequent measurement. The highest value obtained from the available measurements was used for analysis in this study.

### Covariates

This study adjusted for potential confounding variables, including sex, education level, household income, occupation, obesity status, smoking history, alcohol intake, physical activity (PA) level, engagement in MSE, and the presence of diabetes, hypertension, and hypercholesterolemia [8]. Education level was categorized into four groups (elementary, middle school, high school, and undergraduate or higher), and household income was classified into quartiles (low, middle-low, middle-high, and high) based on sex-specific and age-specific equivalized household income as defined in the KNHANES. Occupation was categorized into the following 5 groups [15,16]. In this study, participants who identified as soldiers were excluded from the analysis because of the small sample size (n=47).

(1) White-collar workers: managers, professionals, associate professionals, and office workers; (2) Blue-collar workers: craft workers, machine operators, assemblers, and elementary workers; (3) Green-collar workers: agriculture, forestry, and fisheries workers; (4) Pink-collar workers: service and sales workers; and (5) Not working: individuals who were unemployed or not economically active.

Obesity was defined according to the body mass index (BMI) criteria for the Asia-Pacific region: underweight (BMI<18.5 kg/m^2^), normal weight (18.5 kg/m^2^≤BMI<23.0 kg/m^2^), overweight (23.0 kg/m^2^≤BMI<25.0 kg/m^2^), and obese (BMI≥25.0 kg/m^2^) [17]. Smoking status was categorized into 3 groups: never, past, and current smokers. Alcohol consumption was categorized into 4 groups: non-drinkers (never consumed alcohol), moderate drinkers (up to 2 drinks per day for male and up to 1 drink per day for female), binge drinkers (7 or more drinks for male and 5 or more drinks for female on the same occasion but less than daily), and heavy drinkers (binge drinkers who consumed alcohol on an almost daily basis) [18,19]. PA was assessed using the Global Physical Activity Questionnaire, and the data were processed according to the cleaning protocol provided by the World Health Organization (WHO). MSE was assessed by asking participants how many days per week they engaged in muscle-strengthening activities such as push-ups and pull-ups. The PA guidelines recommended by the WHO were used to classify participants into dichotomous groups: meeting or not meeting the guidelines (PA: ≥150 min/ wk; MSE: ≥2 day/wk). After fasting, participants underwent blood sampling and blood pressure measurement; all participants fasted for at least 8 hours. Diabetes, hypertension, and hypercholesterolemia were each categorized into two groups (presence vs. absence) based on the following criteria: fasting blood glucose ≥126 mg/dL for diabetes; systolic blood pressure ≥140 mmHg or diastolic blood pressure ≥90 mmHg for hypertension; and fasting total cholesterol ≥240 mg/dL for hypercholesterolemia.

### Statistical analysis

Stata version 17 (StataCorp, College Station, TX, USA) was used for all statistical analyses, and statistical significance was set at p-value <0.05. Descriptive statistics were reported as frequencies and weighted percentages for categorical variables. Grip strength was presented with coefficients, standard error (SE), 95% confidence interval (CI), and trends in grip strength over the study period were assessed using multivariable linear regression. For the overall analyses, multivariable linear regression models were adjusted for all listed covariates. For subgroup analyses, the grouping variable (e.g., education, household income, or occupation) was excluded from the adjustment set to avoid over-adjustment, while all other covariates were retained. Grip strength (kg) was modeled as the dependent variable, and survey year (continuous) was treated as the primary independent variable to estimate temporal trends. In subgroup analyses, socioeconomic indicators were used as grouping variables to examine secular patterns across survey years, with all models adjusted as described above except for the subgrouping variable.

### Ethics statement

This study was approved by the Korea National Institute for Bioethics Policy (No. P01-202405-01-005).

## RESULTS

Table 1 summarizes the baseline characteristics of the study population from 2014 to 2022. Over the survey years, the proportion of adults aged ≥50 years increased from 38.4% in 2014 to 48.1% in 2022, and the percentage of participants with an undergraduate or higher education also rose (37.3 to 47.5%). The prevalence of obesity increased (30.7 to 36.9%), whereas the prevalence of current smoking and binge/heavy drinking declined. In parallel, adherence to MSE increased from 25.6% in 2014 to 29.7%.

Table 2 presents the secular trend of grip strength. Mean SE grip strength declined from 35.04±0.21 kg in 2014 to 31.26±0.21 kg in 2018, followed by a rebound to 33.28±0.24 kg in 2022 (p for trend <0.001). Trends for male and female were similar to those observed in the total population (p for trend <0.001). These patterns should be interpreted descriptively, as no formal test for non-linearity was conducted. Sex-specific comparisons demonstrated consistently higher grip strength in male than in female across all survey years. Supplementary Materials 1-4 provide adjusted mean differences, standard errors, 95% CIs, and p-values for each year, showing that differences across sex and socioeconomic groups were statistically significant throughout the study. These results confirm persistent socioeconomic disparities in grip strength alongside temporal changes.

**Figure 2.**
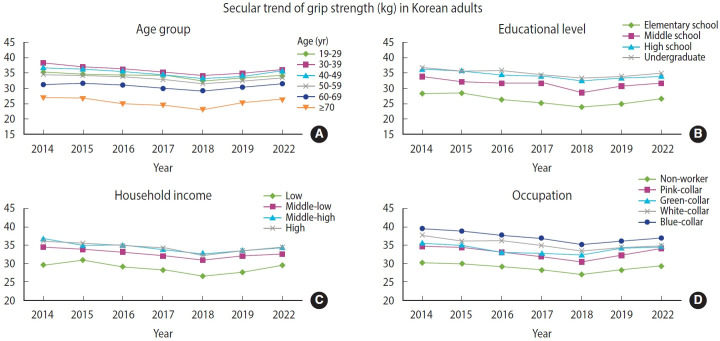
Subgroup trend analyses of grip strength in Korean adults, 2014-2022, stratified by (A) age group, (B) educational level, (C) household income, and (D) occupation. Values are presented as adjusted mean grip strength (kg). Most subgroup trends were statistically significant (p for trend <0.001), except for adults aged ≥70 years (p=0.701) and those in green-collar occupations (p=0.063). Detailed regression results, including coefficients, standard errors, 95% confidence intervals, and exact p-values, are provided in Supplementary Material 5.

Figure 2 illustrates descriptive trends in grip strength stratified by education, household income, and occupation. Across all subgroups, grip strength declined until 2018 and then increased by 2022, showing patterns similar to those in the total population. Participants with higher educational attainment and higher household income consistently demonstrated greater grip strength than those in lower groups, while blue-collar workers had the highest levels among occupational categories. These visual patterns were supported by regression analyses (Supplementary Material 5), which present adjusted mean grip strength for each socioeconomic subgroup by survey year together with regression coefficients, standard errors, 95% CIs, and p for trend values. Most socioeconomic subgroups exhibited statistically significant trends (p for trend <0.001), except for adults aged ≥70 years (p=0.701) and individuals in green-collar occupations (p=0.063).

## DISCUSSION

This study demonstrated secular trends in grip strength among Korean adults using nationally representative KNHANES data from 2014 to 2022. Descriptively, grip strength decreased until 2018 and then increased by 2022. In addition to these temporal patterns, sex differences were consistently observed across all survey years, and these disparities persisted after adjustment for socioeconomic and health-related covariates (Supplementary Material 1). Participants with higher socioeconomic status consistently had greater grip strength than those with lower status.

The findings of this study are consistent with several previous reports. Dodds et al. [8] examined secular trends in grip strength among adults over 50 years in the United Kingdom and noted a slight decline over a decade. Similarly, Kidokoro et al. [9] evaluated changes in Japanese university students from 1973 to 2016, revealing a steady downward trend. Beyond these populations, a study of adults in Shanghai reported a significant decrease in grip strength, particularly among male aged 20-59 years [20], while another investigation in Macao residents aged 6-69 years showed substantial declines between 2001 and 2020 [21]. In Europe, age–period–cohort analyses of older adults in Germany, Sweden, and Spain also demonstrated cohort-related declines in grip strength across successive generations [22]. Taken together, these international findings suggest that declining grip strength has been a common global trend, whereas the recent rebound observed in Korea after 2018 may represent a distinct population pattern that warrants further investigation.

In this study, grip strength decreased until 2018 but increased again by 2022. A possible explanation lies in the parallel patterns of PA and MSE participation during the same period. Adherence to PA guidelines followed a similar trajectory (58.2% in 2014→45.3% in 2018→50.4% in 2022). Additionally, the proportion of participants performing MSE at least twice per week increased from 25.6% in 2014 to 29.7% in 2022. Although causality cannot be inferred from cross-sectional data, the parallel trends of increased PA and MSE adherence alongside higher grip strength suggest a potential relationship that deserves further study. Nevertheless, additional longitudinal analyses with more accumulated data are needed to clarify whether these parallel trends in grip strength, PA, and MSE adherence will persist over time.

It is well established that grip strength decreases progressively with age, a downward trend that was also observed in this study after an initial increase up to the 40s. These results are consistent with previous studies [8,23]. Silverman [23] analyzed secular trends in grip strength among United States and Canadian adults aged 20-79 years and found that grip strength declined by 0.42 kg per year in male and 0.28 kg per year in female. Additionally, a study evaluating changes in grip strength in United Kingdom adults over 50 years indicated that grip strength declines progressively with aging [8]. In this regard, several studies have reported that lower baseline muscle strength increases the risk of heart disease, all-cause mortality, and cardiovascular mortality [24-26]. Furthermore, greater annual declines in muscle strength during the follow-up period are associated with a higher risk of all-cause mortality [27,28]. Therefore, it is crucial to promote engagement in MSE across all age groups to prevent these adverse outcomes and support successful aging.

In this study, participants with higher educational levels exhibited greater grip strength, a finding that is consistent with previous research. A study using nationally representative survey data from Thailand analyzed the relationship between grip strength and education in adults over 60 years and reported that higher educational levels were associated with greater grip strength [14]. Moreover, a study examining the association between grip strength and education in the United Kingdom reported similar results among female; however, no significant difference between educational levels was observed in male [13]. There are several possible explanations for why individuals with higher education levels had higher grip strength. Previous studies have reported that individuals with higher education levels tend to engage in healthier behaviors to maintain better health, such as regular exercise and obesity prevention behaviors [29,30]. Indeed, groups with higher educational attainment in this study performed more PA and had lower obesity rates than those with lower education levels (Supplementary Material 6). Moreover, the completion rate of tertiary education in Korea has increased markedly from 23.8% in 2000 to 52.8% in 2022 [31], indicating a growing number of educated individuals who recognize health maintenance as a critical priority. This trend may result in increasing polarization of grip strength between lower education and higher education groups. Therefore, it is crucial to promote exercise among individuals with lower education levels to help them maintain or improve their health.

This study evaluated differences in grip strength across household income levels and found that participants in higher income groups had greater grip strength. This result is consistent with previous studies. Investigations conducted in the United Kingdom and the United States reported that grip strength was positively correlated with household income [12,13]. In a previous study examining the relationship between socioeconomic status and health behaviors, Pampel et al. [32] showed that lower income groups were more likely to engage in unhealthy behaviors than higher income groups. The authors suggested that individuals in higher socioeconomic positions have better knowledge of the risks associated with unhealthy behaviors, greater access to resources that support healthy behavior, and improved access to medical care, all of which contribute to better health outcomes [32]. Moreover, Strain et al. [33] analyzed the association between income and PA among 327,789 adults from 104 countries, categorizing PA into work/household, transportation, and leisure activities. Their findings indicated that higher income groups engaged more in leisure-time PA (low income: 4.4%; high income: 27.8%) and less in work/household PA (low income: 57.3%; high income: 43.7%) compared with lower-income participants [33]. This suggests that individuals with lower income levels may have less time or opportunity to participate in health-promoting activities because of their greater involvement in work/household PA. Another possible explanation is that educational level may mediate the relationship between income and grip strength. In this study, only 14.1% of individuals in the low household income group had completed undergraduate education, whereas 59.0% of participants in the high household income group had an education level of undergraduate or higher (Supplementary Material 7). Based on these findings, while household income may directly influence grip strength, educational attainment may also serve as a mediating factor in this association.

Our findings showed that participants in blue-collar occupations had the highest grip strength, whereas individuals who were unemployed had the lowest grip strength among the occupational categories. Consistent with this, Mohd Hairi et al. [11] examined the relationship between grip strength and occupation in European adults over 50 years and found that those reporting unemployment or disability had the lowest grip strength. However, in that study, professionals and managers in white-collar occupations had the highest grip strength values. This discrepancy may be explained by several factors highlighted in recent research. First, occupation-related PA plays a significant role in maintaining muscle strength. Blue-collar workers, who routinely perform physically demanding tasks, tend to maintain higher muscle strength through their work activities [33]. White-collar workers, although less physically active during work hours, may engage in PA outside of work more often than individuals not in paid employment, who commonly include the unemployed and retired. In this study, the proportions of participants meeting PA and MSE guidelines were higher among white-collar workers than among the non-working group (53.1 vs. 48.1% for PA; 31.3 vs. 26.3% for MSE; Supplementary Material 8). Second, as mentioned above, socioeconomic status and related health behaviors may also influence grip strength. Non-working groups may have limited access to resources that promote physical health, such as fitness facilities or healthcare services, and may have lower motivation or fewer opportunities to engage in regular PA [32]. Lastly, mental health and social engagement also play important roles. Non-working groups, particularly the unemployed, may experience higher levels of stress, depression, and social isolation, all of which are negatively associated with physical health and muscle strength [34,35].

This study has several strengths. It is the first to examine trends in grip strength according to socioeconomic variables in Korean adults aged ≥19 years. Moreover, the data were obtained from participants selected using a two-stage stratified cluster sampling design, yielding a sample of 34,080 individuals. Therefore, these data are representative of Korean adults, increasing the reliability of the results. However, this study also has several limitations. First, causal relationships between grip strength and socioeconomic variables cannot be inferred because of the cross-sectional study design. Second, analyses of grip strength and covariates were not stratified by sex and age group. Third, grip strength was not measured in KNHANES in 2020 and 2021 due to COVID-19. Therefore, further studies are needed to elucidate the relationship between grip strength and socioeconomic variables stratified by sex and age group, and to determine whether the upward trend in grip strength observed from 2019 onward persisted when including data from subsequent years. Moreover, although standardized protocols were applied for measuring grip strength, differences in examiner technique or device calibration across survey years may have introduced measurement error. Lastly, questionnaires on the presence of some diseases, such as cancer, were not administered in 2022.

In conclusion, although there was an overall decrease in grip strength in both male and female, a slight upward trend was observed from 2019 to 2022. This trend may be partly explained by the gradual increase in the number of individuals engaging in MSE and a similar pattern for PA. In this study, socioeconomic status was associated with grip strength: individuals with lower levels of education and income, as well as those who were unemployed, had lower grip strength. Because these factors are not easily modified over the life course, they may contribute to persistently lower muscle strength. Therefore, this study provides descriptive evidence for identifying socioeconomic groups at risk of low muscle strength, which may help guide future longitudinal research and inform public health strategies.

## Figures and Tables

**Figure f3-epih-47-e2025074:**
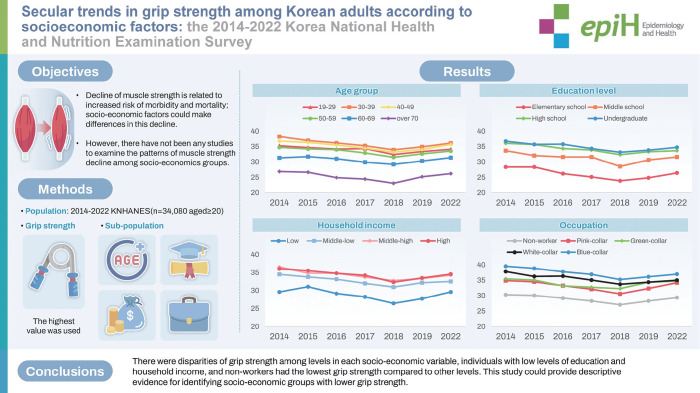


**Table 1.** Characteristics of participants in the 2014-2022 KNHANES

Characteristics	2014	2015	2016	2017	2018	2019	2022
Observation (n)	4,052	4,334	5,096	5,370	5,469	5,482	4,277
Weighted (n)	4,934,843	5,270,776	5,805,061	6,096,129	6,231,816	6,268,174	5,999,799
Sex							
Male	1,756 (50.5)	1,940 (50.4)	2,253 (50.8)	2,402 (50.3)	2,404 (50.1)	2,419 (49.4)	1,874 (50.1)
Female	2,296 (49.5)	2,394 (49.6)	2,843 (49.2)	2,968 (49.7)	3,065 (49.9)	3,063 (50.6)	2,403 (49.9)
Age (yr)							
19-29	500 (19.9)	585 (20.0)	616 (18.5	661 (18.3)	686 (18.1)	675 (18.0)	545 (17.8)
30-39	761 (20.6)	597 (18.3)	949 (19.0)	811 (18.2)	819 (17.9)	824 (17.1)	555 (15.6)
40-49	731 (21.1)	794 (21.0)	993 (21.4)	1,010 (20.6)	1,021 (20.4)	1,026 (20.1)	719 (18.6)
50-59	795 (19.8)	926 (20.2)	924 (19.7)	1,077 (20.1)	1,067 (19.8)	1,063 (20.2)	755 (19.4)
60-69	722 (11.3)	803 (11.9)	845 (12.2)	968 (12.9)	980 (13.2)	986 (14.0)	930 (16.9)
≥70	543 (7.3)	629 (8.7)	769 (9.3)	843 (9.8)	896 (10.6)	908 (10.6)	773 (11.8)
Education							
Elementary school	832 (13.7)	904 (14.6)	1,006 (14.0)	1,072 (13.6)	1,001 (13.1)	948 (11.8)	703 (10.7)
Middle school	444 (9.2)	469 (8.5)	501 (8.5)	537 (8.6)	538 (8.2)	517 (7.8)	348 (6.7)
High school	1,407 (39.8)	1,505 (38.0)	1,650 (36.6)	1,661 (33.3)	1,854 (36.7)	1,852 (36.0)	1,422 (35.1)
Undergraduate	1,367 (37.3)	1,456 (39.0)	1,939 (40.8)	2,100 (44.5)	2,076 (41.9)	2,165 (44.4)	1,804 (47.5)
Household income							
Low	663 (12.2)	728 (13.5)	902 (15.3)	983 (14.4)	951 (14.4)	998 (14.4)	794 (14.9)
Middle-low	1,016 (24.8)	1,062 (22.9)	1,243 (22.9)	1,283 (22.8)	1,318 (24.3)	1,389 (24.7)	1,018 (21.6)
Middle-high	1,214 (31.8)	1,203 (30.4)	1,450 (30.2)	1,494 (30.0)	1,531 (29.7)	1,426 (27.5)	1,179 (30.1)
High	1,159 (31.2)	1,341 (33.2)	1,501 (31.7)	1,610 (32.9)	1,669 (31.5)	1,669 (33.5)	1,286 (33.4)
Occupation							
Non-worker	1,588 (34.9)	1,701 (35.2)	2,033 (36.2)	2,037 (34.1)	2,025 (33.3)	2,124 (34.8)	1,630 (35.1)
Pink-collar	511 (13.9)	560 (13.2)	667 (14.0)	620 (12.2)	780 (15.3)	692 (13.7)	530 (13.4)
Green-collar	218 (3.7)	224 (3.6)	203 (2.5)	260 (3.3)	212 (2.8)	164 (2.1)	191 (2.6)
White-collar	988 (27.7)	1,049 (28.6)	1,248 (27.2)	1,442 (30.9)	1,421 (29.1)	1,418 (29.5)	1,171 (31.1)
Blue-collar	747 (19.8)	800 (19.4)	945 (20.3)	1,011 (19.6)	1,031 (19.5)	1,084 (19.9)	755 (17.9)
Obesity							
Underweight	181 (4.9)	166 (4.6)	195 (4.2)	206 (4.2)	184 (3.5)	218 (4.4)	173 (4.5)
Normal	1,668 (41.6)	1,641 (38.6)	1,929 (37.8)	2,073 (38.7)	2,143 (39.5)	2,144 (38.8)	1,620 (36.2)
Overweight	954 (22.8)	1,049 (23.5)	1,192 (23.2)	1,239 (22.9)	1,238 (21.8)	1,261 (22.6)	965 (22.4)
Obese	1,249 (30.7)	1,478 (33.2)	1,780 (34.8)	1,852 (34.3)	1,904 (35.3)	1,859 (34.2)	1,519 (36.9)
Smoking							
Never	2,470 (57.5)	2,618 (57.1)	3,062 (56.0)	3,249 (57.3)	3,274 (56.8)	3,279 (57.0)	2,592 (57.1)
Past	775 (18.7)	985 (22.3)	1,085 (21.4)	1,161 (21.7)	1,199 (21.9)	1,273 (23.2)	1,036 (25.7)
Current	807 (23.9)	731 (20.6)	949 (22.6)	960 (21.1)	996 (21.3)	930 (19.9)	649 (17.2)
Alcohol							
Non-drinker	1,300 (27.6)	1,370 (27.4)	1,626 (27.0)	1,638 (26.2)	1,679 (27.0)	1,749 (28.4)	1,439 (30.1)
Moderate drinker	1,446 (34.4)	1,567 (35.0)	1,854 (35.4)	2,007 (36.8)	2,040 (36.6)	1,964 (35.7)	1,526 (35.0)
Binge drinker	1,112 (32.5)	1,204 (32.3)	1,340 (31.4)	1,418 (30.6)	1,450 (30.1)	1,507 (31.1)	1,149 (30.6)
Heavy drinker	194 (5.6)	193 (5.3)	276 (6.2)	307 (6.4)	300 (6.4)	262 (4.9)	163 (4.3)
Meets PA guidelines	2,192 (58.2)	2,068 (51.6)	2,314 (48.7)	2,343 (46.9)	2,301 (45.3)	2,380 (45.9)	2,003 (50.4)
Meets MSE guidelines	950 (25.6)	1,070 (27.2)	1,165 (25.2)	1,299 (26.3)	1,344 (26.6)	1,384 (27.5)	1,166 (29.7)
Diabetes	443 (9.0)	459 (8.3)	594 (9.9)	606 (9.3)	650 (10.2)	670 (10.5)	444 (8.8)
Hypertension	524 (11.7)	682 (13.5)	825 (15.4)	811 (13.3)	907 (15.0)	867 (14.0)	546 (11.5)
Hypercholesterolemia	288 (7.0)	406 (9.1)	526 (10.2)	604 (11.1)	549 (10.0)	609 (10.6)	452 (10.9)

Values are presented as number (weighted %); number indicates the unweighted number of participants included in the analysis, while weighted % represents population-level estimates accounting for the KNHANES sampling design. KNHANES, Korea National Health and Nutrition Examination Survey; PA, physical activity; MSE, muscle-strengthening exercise.

**Table 2.** Secular trend of grip strength in the 2014-2022 KNHANES^1^

Sex	n	2014	2015	2016	2017	2018	2019	2022	β	SE	95% CI		p for trend
											LL	UL	
Total	34,080	35.04±0.21	34.31±0.23	33.55±0.19	32.7±0.20	31.26±0.21	32.32±0.19	33.28±0.24	-0.36	0.03	-0.42	-0.30	<0.001
Male	15,048	43.45±0.24	42.63±0.29	42.16±0.22	41.2±0.25	39.67±0.23	40.78±0.24	41.59±0.29	-0.39	0.04	-0.48	-0.31	<0.001
Female	19,032	26.48±0.15	25.83±0.15	24.68±0.16	24.1±0.12	22.82±0.14	24.05±0.13	24.94±0.13	-0.33	0.03	-0.37	-0.28	<0.001

Values are presented as mean±SE. KNHANES, Korea National Health and Nutrition Examination Survey; SE, standard error; CI, confidence interval; LL, lower limit; UL, upper limit.

1 Adjusted mean grip strength (kg) for each socioeconomic subgroup across survey years, estimated from multivariable linear regression models; Models were adjusted for age, sex, education, household income, occupation, body mass index, smoking, alcohol intake, physical activity, muscle-strengthening exercise participation, diabetes, hypertension, and hypercholesterolemia.

## Supplementary materials

Supplementary Material 1. Sex differences in grip strength by survey year, 2014–2022 KNHANES

Supplementary Material 2. Educational differences in grip strength by survey year, 2014–2022 KNHANES

Supplementary Material 3. House income differences in grip strength by survey year, 2014–2022 KNHANES

Supplementary Material 4. Occupational differences in grip strength by survey year, 2014–2022 KNHANES

Supplementary Material 5. Secular trends in grip strength by socioeconomic subgroups (education, household income, and occupation), 2014–2022 KNHANES.

Supplementary Material 6. Characteristics of participants according to educational level in 2014-2022 KHANES

Supplementary Material 7. Characteristics of participants according to household income in 2014-2022 KHANES

Supplementary Material 8. Characteristics of participants according to occupation in 2014-2022 KHANES
