# Supplementary material for: Secular trends in grip strength among Korean adults according to socioeconomic factors: the 2014-2022 Korea National Health and Nutrition Examination Survey
Source: Epidemiol Health. 2025 Dec 16;47:e2025074. doi: 10.4178/epih.e2025074 (PMC12884028; doi:10.4178/epih.e2025074)
Supplement: Supplementary Material 4. — Occupational differences in grip strength by survey year, 2014–2022 KNHANES [file epih-47-e2025074-Supplementary-4.docx]

Supplementary Material 4. Occupational differences in grip strength by survey year, 2014–2022 KNHANES

| **Year** | **Non-worker** | | **Pink-collar** | | **Green-collar** | | **White-collar** | | **Blue-collar** | | **β** | **SE** | **95% CI** | ***p*-value** |
| --- | --- | --- | --- | --- | --- | --- | --- | --- | --- | --- | --- | --- | --- | --- |
|  | **N** | **mean±SE** | **N** | **mean±SE** | **N** | **mean±SE** | **N** | **mean±SE** | **N** | **mean±SE** |  |  |  |  |
| 2014 | 1,588 | 30.28±0.34 | 511 | 34.89±0.50 | 218 | 35.48±0.97 | 988 | 37.83±0.39 | 747 | 39.55±0.47 | 0.54 | 0.07 | 0.40, 0.68 | <.001 |
| 2015 | 1,701 | 30.06±0.31 | 560 | 34.51±0.55 | 224 | 35.08±1.03 | 1,049 | 36.24±0.39 | 800 | 38.87±0.55 | 0.40 | 0.08 | 0.25, 0.55 | <.001 |
| 2016 | 2,033 | 29.21±0.29 | 667 | 33.26±0.47 | 203 | 33.20±0.74 | 1,248 | 36.38±0.39 | 945 | 37.77±0.42 | 0.43 | 0.07 | 0.29, 0.57 | <.001 |
| 2017 | 2,037 | 28.29±0.25 | 620 | 32.12±0.56 | 260 | 32.76±1.10 | 1,442 | 35.08±0.36 | 1,011 | 36.96±0.41 | 0.43 | 0.06 | 0.31, 0.56 | <.001 |
| 2018 | 2,025 | 27.08±0.30 | 780 | 30.56±0.44 | 212 | 32.28±0.72 | 1,421 | 33.67±0.33 | 1,031 | 35.22±0.38 | 0.40 | 0.06 | 0.28, 0.52 | <.001 |
| 2019 | 2,124 | 28.3±0.24 | 692 | 32.31±0.56 | 164 | 34.36±1.40 | 1,418 | 34.36±0.31 | 1,084 | 36.12±0.44 | 0.42 | 0.06 | 0.31, 0.54 | <.001 |
| 2022 | 1,630 | 29.36±0.30 | 530 | 34.21±0.60 | 191 | 34.70±1.10 | 1,171 | 35.02±0.41 | 755 | 37.00±0.47 | 0.42 | 0.07 | 0.28, 0.57 | <.001 |

Values represent adjusted mean differences (occupation groups) in grip strength (kg) for each survey year, estimated from survey-weighted linear regression models. All models were adjusted for age, sex, BMI, smoking, alcohol intake, physical activity, MSE participation, diabetes, hypertension, hypercholesterolemia, and socioeconomic indicators (education and occupation), consistent with the main analyses. Estimates are presented with coefficient (β), standard errors (SE), 95% confidence intervals (CI), and p-values, accounting for the complex sampling design of KNHANES.
