# Supplementary material for: Secular trends in grip strength among Korean adults according to socioeconomic factors: the 2014-2022 Korea National Health and Nutrition Examination Survey
Source: Epidemiol Health. 2025 Dec 16;47:e2025074. doi: 10.4178/epih.e2025074 (PMC12884028; doi:10.4178/epih.e2025074)
Supplement: Supplementary Material 5. — Secular trends in grip strength by socioeconomic subgroups (education, household income, and occupation), 2014–2022 KNHANES. [file epih-47-e2025074-Supplementary-5.docx]

Supplementary Material 5. Secular trends in grip strength by socioeconomic subgroups (education, household income, and occupation), 2014–2022 KNHANES.

|  | **2014** | **2015** | **2016** | **2017** | **2018** | **2019** | **2022** | **β** | **SE** | **95% CI** | ***p* for trend** |
| --- | --- | --- | --- | --- | --- | --- | --- | --- | --- | --- | --- |
|  | **mean±SE** | **mean±SE** | **mean±SE** | **mean±SE** | **mean±SE** | **mean±SE** | **mean±SE** |  |  |  |  |
| **Age group** |  |  |  |  |  |  |  |  |  |  |  |
| 19-29 | 35.24±0.47 | 34.69±0.46 | 34.18±0.51 | 34.33±0.50 | 32.43±0.45 | 33.37±0.49 | 34.09±0.58 | -0.31 | 0.06 | -0.43, -0.20 | <.001 |
| 30-39 | 38.24±0.50 | 36.94±0.52 | 36.15±0.34 | 35.27±0.36 | 33.92±0.41 | 34.88±0.43 | 36.10±0.50 | -0.57 | 0.06 | -0.68, -0.46 | <.001 |
| 40-49 | 36.80±0.39 | 36.35±0.42 | 35.49±0.35 | 34.45±0.41 | 33.23±0.33 | 34.08±0.33 | 35.70±0.42 | -0.40 | 0.05 | -0.50, -0.30 | <.001 |
| 50-59 | 34.73±0.39 | 34.22±0.35 | 34.00±0.38 | 32.92±0.33 | 31.48±0.32 | 32.63±0.35 | 33.55±0.47 | -0.27 | 0.05 | -0.37, -0.18 | <.001 |
| 60-69 | 31.32±0.38 | 31.71±0.39 | 31.06±0.34 | 29.91±0.27 | 29.24±0.32 | 30.29±0.31 | 31.37±0.27 | -0.16 | 0.04 | -0.24, -0.07 | <.001 |
| over 70 | 26.88±0.36 | 26.68±0.42 | 24.9±0.36 | 24.47±0.34 | 23.09±0.35 | 25.18±0.32 | 26.19±0.29 | -0.02 | 0.04 | -0.10, 0.07 | 0.701 |
| **Education** |  |  |  |  |  |  |  |  |  |  |  |
| Elementary school | 28.36±0.39 | 28.36±0.45 | 26.22±0.33 | 25.15±0.33 | 23.86±0.39 | 24.83±0.34 | 26.45±0.34 | -0.17 | 0.05 | -0.26, -0.08 | <.001 |
| Middle school | 33.67±0.59 | 32.05±0.48 | 31.60±0.53 | 31.60±0.53 | 28.54±0.57 | 30.65±0.49 | 31.60±0.61 | -0.33 | 0.07 | -0.46, -0.19 | <.001 |
| High school | 36.06±0.31 | 35.67±0.33 | 34.35±0.31 | 33.88±0.33 | 32.37±0.31 | 33.30±0.26 | 33.68±0.38 | -0.40 | 0.04 | -0.47, -0.32 | <.001 |
| Undergraduate | 36.74±0.35 | 35.68±0.38 | 35.77±0.29 | 34.33±0.30 | 33.14±0.26 | 33.81±0.27 | 34.75±0.32 | -0.36 | 0.04 | -0.45, -0.28 | <.001 |
| **House income** |  |  |  |  |  |  |  |  |  |  |  |
| Low | 29.57±0.44 | 31.03±0.52 | 29.13±0.44 | 28.24±0.41 | 26.45±0.44 | 27.75±0.43 | 29.59±0.45 | -0.23 | 0.05 | -0.33, -0.12 | <.001 |
| Middle-low | 34.51±0.32 | 33.79±0.36 | 33.16±0.34 | 31.96±0.31 | 30.96±0.32 | 32.15±0.32 | 32.50±0.39 | -0.35 | 0.05 | -0.45, -0.25 | <.001 |
| Middle-high | 36.55±0.33 | 34.83±0.37 | 34.79±0.30 | 33.72±0.35 | 32.74±0.37 | 33.35±0.32 | 34.27±0.37 | -0.37 | 0.04 | -0.46, -0.29 | <.001 |
| High | 36.06±0.34 | 35.51±0.38 | 34.8±0.33 | 34.23±0.31 | 32.3±0.30 | 33.46±0.31 | 34.53±0.35 | -0.41 | 0.05 | -0.50, -0.32 | <.001 |
| **Occupation** |  |  |  |  |  |  |  |  |  |  |  |
| Non-worker | 30.28±0.34 | 30.06±0.31 | 29.21±0.29 | 28.29±0.25 | 27.08±0.30 | 28.3±0.24 | 29.36±0.30 | -0.29 | 0.04 | -0.36, -0.22 | <.001 |
| Pink-collar | 34.89±0.50 | 34.51±0.55 | 33.26±0.47 | 32.12±0.56 | 30.56±0.44 | 32.31±0.56 | 34.21±0.60 | -0.39 | 0.06 | -0.50, -0.27 | <.001 |
| Green-collar | 35.48±0.97 | 35.08±1.03 | 33.20±0.74 | 32.76±1.10 | 32.28±0.72 | 34.36±1.40 | 34.70±1.10 | -0.23 | 0.12 | -0.47, 0.01 | 0.063 |
| White-collar | 37.83±0.39 | 36.24±0.39 | 36.38±0.39 | 35.08±0.36 | 33.67±0.33 | 34.36±0.31 | 35.02±0.41 | -0.39 | 0.05 | -0.48, -0.30 | <.001 |
| Blue-collar | 39.55±0.47 | 38.87±0.55 | 37.77±0.42 | 36.96±0.41 | 35.22±0.38 | 36.12±0.44 | 37.00±0.47 | -0.37 | 0.06 | -0.48, -0.25 | <.001 |

Values represent adjusted mean grip strength (kg) for each socioeconomic subgroup across survey years, estimated from multivariable linear regression models. Models were adjusted for age, sex, BMI, smoking, alcohol intake, physical activity, MSE participation, diabetes, hypertension, and hypercholesterolemia, except for the subgrouping variable. Results are presented with coefficients (β**)**, standard errors (SE), 95% confidence intervals (CI), and *p* for trend.
