# Supplementary material for: Secular trends in grip strength among Korean adults according to socioeconomic factors: the 2014-2022 Korea National Health and Nutrition Examination Survey
Source: Epidemiol Health. 2025 Dec 16;47:e2025074. doi: 10.4178/epih.e2025074 (PMC12884028; doi:10.4178/epih.e2025074)
Supplement: Supplementary Material 6. — Characteristics of participants according to educational level in 2014-2022 KHANES [file epih-47-e2025074-Supplementary-6.docx]

Supplementary Material 6. Characteristics of participants according to educational level in 2014-2022 KHANES

|  | **Elementary school** | | **Middle school** | | **High school** | | **Undergraduate** | |
| --- | --- | --- | --- | --- | --- | --- | --- | --- |
|  | **N** | **weighted %** | **N** | **weighted %** | **N** | **weighted %** | **N** | **weighted %** |
| Observation | 6,466 |  | 3,354 |  | 11,351 |  | 12,909 |  |
| Weighted number | 5,287,028 |  | 3,326,482 |  | 14,767,050 |  | 17,226,039 |  |
| **Sex** |  |  |  |  |  |  |  |  |
| Men | 2,064 | 33.3 | 1,509 | 48.6 | 5,293 | 52.4 | 6,182 | 53.8 |
| Women | 4,402 | 66.7 | 1,845 | 51.4 | 6,058 | 47.6 | 6,727 | 46.2 |
| **age group** |  |  |  |  |  |  |  |  |
| 19-29 | 16 | 0.6 | 48 | 2.7 | 2,308 | **28.1** | 1,896 | **19.1** |
| 30-39 | 33 | 0.9 | 105 | 4.3 | 1,332 | **12.5** | 3,846 | **30.7** |
| 40-49 | 84 | 2.1 | 235 | 10.3 | 2,454 | **21.9** | 3,521 | **26.7** |
| 50-59 | 753 | **16.0** | 1,015 | **35.7** | 2,751 | 23.0 | 2,088 | 15.4 |
| 60-69 | 2,284 | **35.1** | 1,227 | **30.6** | 1,664 | 10.3 | 1,056 | 5.8 |
| over 70 | 3,293 | **45.5** | 724 | **16.3** | 842 | 4.3 | 502 | 2.3 |
| **House income** |  |  |  |  |  |  |  |  |
| Low | 3,161 | **46.0** | 815 | 22.8 | 1,422 | 12.0 | 621 | 4.7 |
| Middle-low | 1,885 | **29.2** | 1,132 | **32.6** | 3,020 | 26.0 | 2,292 | 17.7 |
| Middle-high | 927 | 16.2 | 827 | **27.1** | 3,618 | **32.4** | 4,125 | **32.4** |
| High | 493 | 8.6 | 580 | 17.6 | 3,291 | **29.6** | 5,871 | **45.2** |
| **Occupation** |  |  |  |  |  |  |  |  |
| Non-worker | 3,637 | **54.5** | 1,408 | 38.6 | 4,416 | 37.5 | 3,677 | 25.7 |
| Pink-collar | 525 | 8.9 | 502 | 15.6 | 2,077 | 18.7 | 1,256 | 10.5 |
| Green-collar | 735 | 9.7 | 283 | 6.6 | 311 | 2.0 | 143 | 0.9 |
| White-collar | 35 | 0.6 | 110 | 3.3 | 1,865 | **16.9** | 6,727 | **53.6** |
| Blue-collar | 1,534 | 26.3 | 1,051 | **35.9** | 2,682 | **24.9** | 1,106 | 9.5 |
| **Obesity** |  |  |  |  |  |  |  |  |
| Underweight | 144 | 2.1 | 75 | 2.2 | 459 | 4.8 | 645 | 5.0 |
| Normal | 2,022 | 30.9 | 1,129 | 33.5 | 4,552 | **39.8** | 5,515 | **41.2** |
| Overweight | 1,681 | 26.1 | 866 | 26.2 | 2,595 | 22.4 | 2,756 | 21.2 |
| Obese | 2,619 | **40.9** | 1,284 | **38.1** | 3,745 | 33.0 | 3,993 | 32.7 |
|  | **Elementary school** | | **Middle school** | | **High school** | | **Undergraduate** | |
|  | **N** | **weighted %** | **N** | **weighted %** | **N** | **weighted %** | **N** | **weighted %** |
| **Smoking** |  |  |  |  |  |  |  |  |
| Never | 4,464 | 67.5 | 1,892 | 53.0 | 6,499 | 54.7 | 7,689 | 56.4 |
| Past | 1,268 | 19.4 | 862 | 25.6 | 2,362 | 20.1 | 3,022 | 24.3 |
| Current | 734 | 13.2 | 600 | 21.4 | 2,490 | 25.2 | 2,198 | 19.3 |
| **Alcohol** |  |  |  |  |  |  |  |  |
| Non-drinker | 3,455 | 52.0 | 1,354 | 36.9 | 3,028 | 24.6 | 2,964 | 21.1 |
| Moderate drinker | 1,935 | 29.0 | 1,069 | 31.4 | 4,064 | 33.9 | 5,336 | 39.9 |
| Binge drinker | 791 | 13.9 | 700 | 23.4 | 3,599 | 35.2 | 4,090 | 34.5 |
| Heavy drinker | 285 | 5.0 | 231 | 8.3 | 660 | 6.3 | 519 | 4.6 |
| **Meeting PA guideline** | 1,942 | 31.2 | 1,277 | 39.1 | 5,634 | **52.6** | 6,748 | **53.9** |
| **Meeting MSE guideline** | 834 | 13.4 | 660 | 19.5 | 3,015 | **28.5** | 3,869 | **31.1** |
| **Diabetes** | 1,382 | 20.9 | 604 | 17.4 | 1,119 | 8.4 | 761 | 5.3 |
| **Hypertension** | 1,642 | 24.3 | 669 | 19.7 | 1,488 | 11.8 | 1,363 | 10.6 |
| **Hypercholesterolemia** | 643 | 10.4 | 350 | 10.8 | 1,134 | 9.5 | 1,307 | 10.0 |

Values are presented as Number (N) and weighted %. N indicates the unweighted number of participants included in the analysis, while weighted % represents population-level estimates accounting for the KNHANES sampling design.
