# Supplementary material for: Secular trends in grip strength among Korean adults according to socioeconomic factors: the 2014-2022 Korea National Health and Nutrition Examination Survey
Source: Epidemiol Health. 2025 Dec 16;47:e2025074. doi: 10.4178/epih.e2025074 (PMC12884028; doi:10.4178/epih.e2025074)
Supplement: Supplementary Material 7. — Characteristics of participants according to household income in 2014-2022 KHANES [file epih-47-e2025074-Supplementary-7.docx]

Supplementary Material 7. Characteristics of participants according to household income in 2014-2022 KHANES

|  | **Low** | | **Middle-low** | | **Middle-high** | | **High** | |
| --- | --- | --- | --- | --- | --- | --- | --- | --- |
|  | **N** | **weighted %** | **N** | **weighted %** | **N** | **weighted %** | **N** | **weighted %** |
| Observation | 6,019 |  | 8,329 |  | 9,497 |  | 10,235 |  |
| Weighted number | 5,769,523 |  | 9,512,038 |  | 12,128,162 |  | 13,196,876 |  |
| **Sex** |  |  |  |  |  |  |  |  |
| Men | 2,415 | 43.6 | 3,641 | 49.0 | 4,257 | 51.6 | 4,735 | 52.7 |
| Women | 3,604 | 56.4 | 4,688 | 51.0 | 5,240 | 48.4 | 5,500 | 47.3 |
| **Age group** |  |  |  |  |  |  |  |  |
| 19-29 | 490 | 16.3 | 948 | 17.6 | 1,304 | 19.0 | 1,526 | 19.9 |
| 30-39 | 238 | 5.3 | 1,277 | 18.5 | 1,999 | **22.8** | 1,802 | 18.8 |
| 40-49 | 107 | 9.2 | 1,333 | 18.5 | 2,137 | **23.6** | 2,417 | **23.8** |
| 50-59 | 646 | 13.4 | 1,409 | 17.6 | 1,828 | 18.7 | 2,724 | **25.5** |
| 60-69 | 1,410 | **19.6** | 1,952 | 17.1 | 1,529 | 11.5 | 1,343 | 9.4 |
| over 70 | 2,828 | **36.2** | 1,410 | 10.6 | 700 | 4.5 | 423 | 2.6 |
| **Education** |  |  |  |  |  |  |  |  |
| Elementary school | 3,161 | **42.2** | 1,885 | 16.3 | 927 | 7.1 | 493 | 3.4 |
| Middle school | 815 | 13.1 | 1,132 | 11.4 | 827 | 7.4 | 580 | 4.4 |
| High school | 1,422 | 30.7 | 3,020 | **40.3** | 3,618 | **39.5** | 3,291 | **33.2** |
| Undergraduate | 621 | 14.1 | 2,292 | **32.0** | 4,125 | **46.0** | 5,871 | **59.0** |
| **Occupation** |  |  |  |  |  |  |  |  |
| Non-worker | 3,797 | **60.6** | 3,462 | 38.5 | 3,100 | 29.7 | 2,779 | 25.5 |
| Pink-collar | 481 | 9.7 | 1,147 | 14.8 | 1,409 | 15.4 | 1,323 | 13.0 |
| Green-collar | 479 | 6.2 | 503 | 4.4 | 260 | 1.6 | 230 | 1.6 |
| White-collar | 261 | 6.5 | 1,303 | 18.8 | 2,700 | **30.7** | 4,473 | **45.2** |
| Blue-collar | 1,001 | 17.0 | 1,914 | 23.6 | 2,028 | 22.6 | 1,430 | 14.7 |
| **Obesity** |  |  |  |  |  |  |  |  |
| Underweight | 223 | 4.2 | 315 | 4.5 | 374 | 4.1 | 411 | 4.4 |
| Normal | 2,179 | 37.4 | 3,063 | 37.1 | 3,727 | 38.6 | 4,249 | 40.4 |
| Overweight | 1,421 | 22.5 | 1,970 | 23.2 | 2,111 | 22.0 | 2,396 | 23.1 |
| Obese | 2,196 | 35.9 | 2,981 | 35.2 | 3,285 | 35.3 | 3,179 | 32.1 |
|  | **Low** | | **Middle-low** | | **Middle-high** | | **High** | |
|  | **N** | **weighted %** | **N** | **weighted %** | **N** | **weighted %** | **N** | **weighted %** |
| **Smoking** |  |  |  |  |  |  |  |  |
| Never | 3,698 | 59.6 | 4,961 | 56.8 | 5,624 | 55.2 | 6,261 | 57.6 |
| Past | 1,339 | 20.8 | 1,892 | 22.4 | 2,011 | 22.0 | 2,272 | 22.9 |
| Current | 982 | 19.6 | 1,476 | 20.8 | 1,862 | 22.9 | 1,702 | 19.5 |
| **Alcohol** |  |  |  |  |  |  |  |  |
| Non-drinker | 2,969 | 43.7 | 2,857 | 30.7 | 2,607 | 24.9 | 2,368 | 21.1 |
| Moderate drinker | 1,765 | 29.7 | 2,933 | 34.1 | 3,576 | 36.2 | 4,130 | 38.8 |
| Binge drinker | 1,021 | 21.5 | 2,074 | 29.1 | 2,839 | 33.5 | 3,246 | 34.8 |
| Heavy drinker | 264 | 5.2 | 465 | 6.2 | 475 | 5.4 | 491 | 5.4 |
| **Meeting PA guideline** | 2,126 | 40.1 | 3,654 | 47.3 | 4,547 | 50.2 | 5,274 | 53.8 |
| **Meeting MSE guideline** | 1,027 | 19.6 | 1,881 | 24.2 | 2,413 | 27.4 | 3,057 | 31.6 |
| **Diabetes** | 1,157 | 16.8 | 1,064 | 10.8 | 872 | 7.9 | 773 | 6.7 |
| **Hypertension** | 1,342 | 20.0 | 1,331 | 14.4 | 1,238 | 12.0 | 1,251 | 11.6 |
| **Hypercholesterolemia** | 594 | 10.0 | 820 | 9.6 | 957 | 10.2 | 1,063 | 10.0 |

Values are presented as Number (N) and weighted %. N indicates the unweighted number of participants included in the analysis, while weighted % represents population-level estimates accounting for the KNHANES sampling design.
