# Supplementary material for: Secular trends in grip strength among Korean adults according to socioeconomic factors: the 2014-2022 Korea National Health and Nutrition Examination Survey
Source: Epidemiol Health. 2025 Dec 16;47:e2025074. doi: 10.4178/epih.e2025074 (PMC12884028; doi:10.4178/epih.e2025074)
Supplement: Supplementary Material 8. — Characteristics of participants according to occupation in 2014-2022 KHANES [file epih-47-e2025074-Supplementary-8.docx]

Supplementary Material 8. Characteristics of participants according to occupation in 2014-2022 KHANES

|  | **Non-worker** | | | **Pink-collar** | | | | **Green-collar** | | | **White-collar** | | | | **Blue-collar** | | | |
| --- | --- | --- | --- | --- | --- | --- | --- | --- | --- | --- | --- | --- | --- | --- | --- | --- | --- | --- |
|  | **N** | | **weighted %** | **N** | | **weighted %** | | **N** | | **weighted %** | **N** | | **weighted %** | | **N** | | **weighted %** |  |
| Observation | 13,138 | |  | 4,360 | |  | | 1,472 | |  | 8,737 | |  | | 6,373 | |  |  |
| Weighted number | 14,116,498 | |  | 5,552,568 | |  | | 1,174,360 | |  | 11,863,284 | |  | | 7,899,889 | |  |  |
| **Sex** |  | |  |  | |  | |  | |  |  | |  | |  | |  |  |
| Men | 4,128 | | 34.5 | 1,528 | | 43.5 | | 905 | | 65.8 | 4,438 | | 56.9 | | 4,049 | | 70.6 |  |
| Women | 9,010 | | 65.5 | 2,832 | | 56.5 | | 567 | | 34.2 | 4,299 | | 43.1 | | 2,324 | | 29.4 |  |
| **Age group** |  | |  |  | |  | |  | |  |  | |  | |  | |  |  |
| 19-29 | 1,798 | | 22.6 | 691 | | 22.0 | | 6 | | 1.2 | 1,401 | | 20.1 | | 372 | | 9.4 |  |
| 30-39 | 1,474 | | 13.1 | 575 | | 14.5 | | 41 | | 4.3 | 2,490 | | **29.3** | | 736 | | **14.3** |  |
| 40-49 | 1,432 | | 12.2 | 955 | | 22.7 | | 85 | | 9.4 | 2,622 | | **29.0** | | 1,200 | | **22.5** |  |
| 50-59 | 1,765 | | 14.4 | 1,261 | | 27.4 | | 303 | | 26.0 | 1,533 | | **16.5** | | 1,745 | | **28.6** |  |
| 60-69 | 2,882 | | 17.4 | 700 | | 11.0 | | 565 | | 34.8 | 560 | | 4.4 | | 1,527 | | 17.7 |  |
| over 70 | 3,787 | | 20.3 | 178 | | 2.4 | | 472 | | 24.3 | 131 | | 0.9 | | 793 | | 7.6 |  |
| **Education** |  | |  |  | |  | |  | |  |  | |  | |  | |  |  |
| Elementary school | 3,637 | | 20.4 | 525 | | 8.4 | | 735 | | **43.8** | 35 | | 0.3 | | 1,534 | | 17.6 |  |
| Middle school | 1,408 | | 9.1 | 502 | | 9.3 | | 283 | | 18.7 | 110 | | 0.9 | | 1,051 | | 15.1 |  |
| High school | 4,416 | | 39.2 | 2,077 | | **49.7** | | 311 | | 25.1 | 1,865 | | 21.0 | | 2,682 | | **46.6** |  |
| Undergraduate | 3,677 | | 31.3 | 1,256 | | 32.6 | | 143 | | 12.4 | 6,727 | | **77.8** | | 1,106 | | 20.6 |  |
| **House income** |  | |  |  | |  | |  | |  |  | |  | |  | |  |  |
| Low | 3,797 | | 24.8 | 481 | | 10.1 | | 479 | | 30.3 | 261 | | 3.2 | | 1,001 | | 12.4 |  |
| Middle-low | 3,462 | | 25.9 | 1,147 | | 25.3 | | 503 | | 35.5 | 1,303 | | 15.1 | | 1,914 | | 28.4 |  |
| Middle-high | 3,100 | | 25.5 | 1,409 | | 33.7 | | 260 | | 16.3 | 2,700 | | 31.4 | | 2,028 | | 34.7 |  |
| High | 2,779 | | 23.8 | 1,323 | | 30.9 | | 230 | | 17.9 | 4,473 | | 50.3 | | 1,430 | | 24.5 |  |
| **Obesity** |  | |  |  | |  | |  | |  |  | |  | |  | |  |  |
| Underweight | 614 | | 5.6 | 163 | | 4.1 | | 28 | | 1.6 | 377 | | 4.3 | | 141 | | 2.5 |  |
| Normal | 5,354 | | 41.9 | 1,645 | | 37.8 | | 476 | | 31.7 | 3,575 | | 39.4 | | 2,168 | | 33.6 |  |
| Overweight | 2,912 | | 21.1 | 1,027 | | 23.2 | | 389 | | 26.7 | 1,971 | | 22.7 | | 1,599 | | 24.8 |  |
| Obese | 4,258 | | 31.5 | 1,525 | | 34.9 | | 579 | | 40.1 | 2,814 | | 33.6 | | 2,465 | | 39.1 |  |
|  | | **Non-worker** | | | **Pink-collar** | | | | **Green-collar** | | **White-collar** | | | **Blue-collar** | | | | |
|  | | **N** | **weighted %** | | **N** | | **weighted %** | | **N** | **weighted %** | **N** | **weighted %** | | **N** | | **weighted %** | |  |
| **Smoking** | |  |  | |  | |  | |  |  |  |  | |  | |  | |  |
| Never | | 9,179 | 69.5 | | 2,759 | | 58.1 | | 720 | 45.3 | 5,045 | 54.1 | | 2,841 | | 39.7 | |  |
| Past | | 2,481 | 17.4 | | 725 | | 18.0 | | 466 | 31.5 | 2,061 | 24.9 | | 1,781 | | 28.3 | |  |
| Current | | 1,478 | 13.0 | | 876 | | 23.9 | | 286 | 23.2 | 1,631 | 21.0 | | 1,751 | | 32.0 | |  |
| **Alcohol** | |  |  | |  | |  | |  |  |  |  | |  | |  | |  |
| Non-drinker | | 5,516 | 37.8 | | 1,116 | | 22.9 | | 607 | 39.1 | 1,698 | 18.0 | | 1,864 | | 25.8 | |  |
| Moderate drinker | | 4,872 | 37.5 | | 1,558 | | 33.9 | | 462 | 28.2 | 3,465 | 38.0 | | 2,047 | | 31.0 | |  |
| Binge drinker | | 2,338 | 21.2 | | 1,411 | | 36.1 | | 287 | 23.3 | 3,147 | 38.7 | | 1,997 | | 35.2 | |  |
| Heavy drinker | | 412 | 3.4 | | 275 | | 7.0 | | 116 | 9.5 | 427 | 5.3 | | 462 | | 8.1 | |  |
| **Meeting PA guideline** | | 5,750 | 48.1 | | 2,089 | | 50.8 | | 467 | 36.0 | 4,479 | 53.1 | | 2,816 | | 46.5 | |  |
| **Meeting MSE guideline** | | 3,119 | 26.3 | | 1,036 | | 26.3 | | 170 | 13.9 | 2,621 | 31.3 | | 1,432 | | 23.7 | |  |
| **Diabetes** | | 1,792 | 11.1 | | 407 | | 8.3 | | 260 | 17.4 | 531 | 5.5 | | 876 | | 12.1 | |  |
| **Hypertension** | | 2,111 | 13.5 | | 582 | | 12.2 | | 313 | 19.9 | 944 | 10.9 | | 1,212 | | 17.7 | |  |
| **Hypercholesterolemia** | | 1,324 | 10.0 | | 472 | | 10.0 | | 120 | 9.1 | 857 | 9.8 | | 661 | | 10.2 | |  |

Values are presented as Number (N) and weighted %. N indicates the unweighted number of participants included in the analysis, while weighted % represents population-level estimates accounting for the KNHANES sampling design.
